# Association between *APOE* polymorphisms and lipid profile in Mexican Amerindian population

**DOI:** 10.1002/mgg3.958

**Published:** 2019-09-26

**Authors:** José J. Martínez‐Magaña, Alma D. Genis‐Mendoza, Carlos A. Tovilla‐Zarate, Thelma B. González‐Castro, Isela Esther Juárez-Rojop, Yazmín Hernández‐Díaz, Angélica G. Martinez‐Hernandez, Humberto Garcia‐Ortíz, Lorena Orozco, María L. López‐Narvaez, Humberto Nicolini

**Affiliations:** ^1^ National Institute of Genomic Medicine (Instituto Nacional de Medicina Genómica INMEGEN) Laboratory of Genomics of Psychiatric Diseases, Neurodegenerative and Addictions Ministry of Health Mexico City Mexico; ^2^ Comalcalco Multidisciplinary Academic Division Autonomous Juárez University of Tabasco (Universidad Juárez Autónoma de Tabasco) Comalcalco Tabasco Mexico; ^3^ Multidisciplinary Academic Division of Jalpa de Méndez Autonomous Juárez University of Tabasco (Universidad Juárez Autónoma de Tabasco) Jalpa de Méndez Tabasco Mexico; ^4^ Academic Division of Health Sciences Autonomous Juárez University of Tabasco (Universidad Juárez Autónoma de Tabasco) Villahermosa Tabasco Mexico; ^5^ National Institute of Genomic Medicine (INMEGEN) Laboratory of Immunogenomics and Metabolic Diseases Ministry of Health Mexico City Mexico; ^6^ General Hospital of Yajalon Ministry of Health Yajalon Chiapas Mexico

**Keywords:** *APOE* gene, body mass index, cholesterol, Mexican population, triglyceride

## Abstract

**Background:**

Apolipoprotein E (ApoE) is a glycoprotein that plays an important role in lipid homeostasis at both cerebral and systemic levels. Moreover, the differential distribution of *APOE* gene alleles among different populations, means that ApoE isoforms could have different effects on lipids metabolism. The present study aims to evaluate the relationship between *APOE* gene alleles and the lipid profile in a Mexican Amerindian (MA) population.

**Methods:**

This study included 1997 MA individuals of different ethnicities distributed throughout different states of Mexico. All individuals underwent anthropometric measurements as well as laboratory tests including fasting glucose (FG), total cholesterol (TC), triglycerides, low‐density lipoprotein cholesterol (LDL‐C), and high‐density lipoprotein cholesterol (HDL‐C). TaqMan^®^ probe genotyping assays were used to genotype *APOE*. The Kruskal–Wallis test was performed to determine the correlation between *APOE* gene alleles and genotypes and the biochemical variables measured.

**Results:**

Among the biochemical variables analyzed, only the HDL‐C and LDL‐C levels showed statistical differences (*p*‐value < .05) between individuals carrying different *APOE* alleles. For HDL‐C, individuals carrying the E2 allele had higher HDL‐C levels, followed by individuals carrying the E3 allele and carriers of the E4 allele presented the lowest levels of HDL‐C (E2 > E3 > E4). This relationship was inversed for LDL‐C levels (E2 < E3 < E4). Nevertheless, the difference of HDL‐C levels between *APOE‐E3* and *APOE‐E4* carriers remained only in obese individuals.

**Conclusions:**

Our results suggest that *APOE* gene genotypes play an important role in the differential modulation of lipid profiles in the MA population with obesity.

## 1 INTRODUCTION

Apolipoprotein E (ApoE) is a 34‐kDa glycoprotein with 299 amino acid residues that plays a key role in lipid metabolism (Mahley, 1988; Smelt & de Beer, 2004). ApoE is involved in the reverse transport of cholesterol and clearance of very low‐density lipoprotein (LDL) remnants (Greenow, Pearce, & Ramji, 2005; Phillips, 2014). ApoE in humans is encoded by a 3647‐bp gene found in the 19q13.32 region (Nguyen, Dhanasekaran, Phillips, & Lund‐Katz, 2009). The three primary ApoE protein isoforms are originated from two non‐synonymous single nucleotide polymorphisms (SNPs) which are located in the coding region of the LDL receptor binding domain of the protein (rs429358 and rs7412); the two SNPs are in high linkage disequilibrium (Saito et al., 2003; Takei et al., ). The rs429358 polymorphism promotes a shift of a cysteine residue (C) to an arginine residue (R) at position 112 of the protein (p.C112R), whereas the rs7412 promotes a change of an R residue to a C residue at position 158 of the protein (p.R158C) (Saito et al., 2003). Three different gene alleles and three protein isoforms arise from the combination of the two SNPs and are commonly referred to as E2, E3, and E4 (Nguyen et al., 2009; Suarez & Schonfeld, 1981).

The *APOE* E2 allele encodes a protein with a Cys residue at both positions of the protein, the E3 allele encodes a Cys residue at position 112 and an Arg residue at position 158 and the E4 allele encodes an Arg protein at both positions (Nguyen et al., 2010; Suarez & Schonfeld, 1981). The three ApoE isoforms are originated from the substitution of these amino acid residues and lead to differential functions in lipid metabolism (Boerwinkle, Brown, Sharrett, Heiss, & Patsch, 1994; Boerwinkle & Utermann, 1988; Gregg & Brewer, 1988; Gregg et al., 1986; Hauser, Narayanaswami, & Ryan, 2011; Villeneuve, Brisson, & Gaudet, 2015). Additionally, each isoform of ApoE conveys a different risk of some diseases, such as Alzheimer's disease (AD) (Chen, Baum, Ng, Chan, & Pang, 1999) schizophrenia (Vila‐Rodriguez, Honer, Innis, Wellington, & Beasley, 2011), osteoporosis (Singh, Singh, Singh, Juneja, & Kaur, 2010), or arteriosclerosis, which could be due the different functions and structures of the ApoE isoforms (Mahley, Weisgraber, & Huang, 2009; Ray, Ahalawat, & Mondal, 2017).

Due to a different distribution of *APOE* alleles among different ethnic populations, ApoE isoforms may also have a differential lipid metabolism which results in different lipid profiles among populations. For instance, it is well known that LDL cholesterol (LDL‐C), high‐density lipoprotein cholesterol (HDL‐C), and total cholesterol (TC) levels in individuals with a higher indigenous component differ significantly compared with the levels observed in individuals of African (AFR) origin, and it seems that a differential lipid modulation could be influenced by population‐specific genetic and epigenetic factors, such as differential *APOE* alleles distribution (Huebbe & Rimbach, 2017; Rodriguez et al., 2002). The current Mexican population is considered mestizo (MM) which comes from a mixture of three main populations: Mexican Amerindian (MA), Caucasian and AFR. Thus, the MM genome is a conjunction of regions of the genome derived from these different origins (Silva‐Zolezzi et al., 2009). There the importance of understanding how the *APOE* alleles differentially modulate lipid profile in the main populations that originated the MM, in order to better understand the relationship between *APOE* and lipid profile. Hence, the present study aims to evaluate the relationship between biochemical variables (FG, TC, triglycerides [TGs], LDL‐C, and HDL‐C) and *APOE* alleles in a sample of MA individuals.

## 2 MATERIALS AND METHODS

### 2.1 Ethical compliance

This protocol was approved by the ethics and research committees of the National Institute of Genomic Medicine (Approval number: INMG/DI/149/2014). All individuals signed a letter of informed consent.

### 2.2 Study population

The study included a total of 1997 individuals of Mexican native descent from different regions of Mexico. Recruited individuals belonged to the Mexican indigenous cohort of a metabolic study (MAIS). Considerations for the inclusion of individuals had been previously reported (Contreras‐Cubas et al., 2016).

### 2.3 Sociodemographic and biochemical variables

Information regarding age, gender, and education of every individual was collected through a structured questionnaire. Height in metres and weight in kilograms were measured using the Tanita^®^ digital scale (Tanita Corporation, Clearbrook) with the stadiometer adjusted to 1‐mm pressure. The body mass index (BMI) was calculated by dividing the weight in kilograms by the square of the height in metres. Biochemical tests, such as FG, TGs, HDL‐C, LDL‐C, and TC, were determined using the Cholestec LDX system^®^ (Bio‐Rad).

### 2.4 Genotyping

Genotyping of rs7412 and rs429358 polymorphisms on *APOE* (NC_000019.9) was performed with allelic discrimination assays using Taqman^®^ probes (C_3084793_20 and C_904973_10, respectively, Applied Biosystems). The thermocycling conditions used for each test were established by the supplier. The thermocycling and allelic discrimination were performed using the 7900 real‐time equipment with the SDS v2.1 software (Applied Biosystems). As a quality control for genotyping, 20% of the samples were genotyped in duplicate.

### 2.5 Statistical analysis

Data are presented as numbers with frequencies and percentages for categorical variables. The demographic description (years of age, years of school), anthropometric (BMI) and clinic characteristics (glucose, cholesterol, high‐density lipoprotein, LDL, and TG) are presented by means and standard deviations as continues variables.

Because different ApoE isoforms have non‐identical functions in relation to lipid metabolism (Mahley et al., 2009), we performed two different analyses. First, MA individuals were divided into two groups according with the *APOE* alleles carried (i.e., carrying E2+, E3/E3 homozygotes, and carrying E4+). Next, we divided the population into five groups according with the *APOE* genotypes, the groups were as follows: (a) individuals with E2/E4 genotype, (b) individuals with E2/E3 genotype, (c) individuals homozygous for E3 allele, (d) individuals with E3/E4 genotype (e) individuals homozygous for E4 allele. For continuous variables we tested for normality in all the variables using Shapiro–Wilk normality test and all the variables had a non‐normal distribution (*p*‐value < .0001). The continuous variables are presented as medians and interquartile distance, while categorical variables are presented as total individuals and percentages. Next, in order to explore differences between the groups we performed the Kruskal–Wallis test. A *p*‐value lower than .05 was considered significant. All statistical analyses were performed using the free R software.

## 3 RESULTS

### 3.1 Sociodemographic variables

The majority of MA individuals were women (68.5%) with a mean age of 51.19 years. Regarding their educational level, 23.79% (*N* = 470) did not have any formal education, and 76.21% (*N* = 1679) had only 3 years of education. In relation to the BMI, 32.65% (*n* = 652) of the total sample presented normal weight 798 individuals (39.96%) were overweight and 547 individuals (27.39%) were obese. The summary of sociodemographic and biochemical variables of the MA population is shown in Table 1.

**Table 1:** Sociodemographic and biochemical characteristics of 1997 Mexican Amerindian (MA) individuals

Sociodemographic	
Gender^a^	
Men	630 (31.5%)
Women	1,367 (68.5%)
Age (years)	50 (39–63)
Total public education (years)	1.0 (0.0–2.0)
Body mass index (BMI, kg/m^2^)	27.1 (24.2–30.4)
Normal weight	652 (32.65%)
Overweight	798 (39.96%)
Obese	547 (27.39%)
Biochemical	
Fasting glucose (FG, mg/dL)^b^	92.0 (82.0–105.0)
Total cholesterol (TC, mg/dL)	178.0 (154.0–205.0)
Triglycerides (TG, mg/dL)	168.5 (121.0–239.2)
High‐density cholesterol (HDL‐C, mg/dL)	38.00 (31.0–46.0)
Low‐density cholesterol (LDL‐C, mg/dL)	101.00 (82.0–122.0)
APOE	
Genotype frequencies	
E2/E4	6 (0.30%)
E2/E3	15 (0.75%)
E3/E3	1,589 (79.57%)
E3/E4	354 (17.73%)
E4/E4	33 (1.65%)
Allelic frequencies	
E2	21 (0.53%)
E3	3,547 (88.81%)
E4	426 (10.67%)

a Results by gender, normal weight, overweight, obese, and genotype frequencies are of the total individuals (percentages in parentheses).

b For the BMI and biochemical variables, the results are reported as medians (interquartile range in parentheses).

### 3.2 Association of APOE polymorphisms with lipid and glucose profiles

The analysis of *APOE* alleles distribution showed that *APOE‐E3* was the most frequent (88.81%), followed by *APOE‐E4* (10.67%) and the *APOE‐E2* allele was the least frequent (0.53%). The most frequent genotype was *E3* homozygote (79.57%). With regards of *APOE‐E2* allele, no *E2* homozygote was found, only as heterozygote E2/E4 and E2/E3 (*APOE‐E2+*). For individuals carrying the *APOE‐E4* allele, the most frequent form was heterozygote E3/E4 (17.73%); homozygote E4 represented 1.65%, and overall, individuals carrying *APOE‐E4* allele represented 19.38% in this MA sample (Table 1).

First, we analyzed differences observed on biochemical tests in correlation to different *APOE* allele carriers (i.e. comparing E2+, E3/E3, and E4+) (Table 2). Individuals with E2+ presented significantly higher HDL‐C values (median: 41.0 [38.3–51.8]) than individuals with E3/E3 genotype (median: 38.0 [31.6–46.0]), and individuals carrying the E4 allele (median: 36.0 [30.0–44.0]). Levels of LDL‐C in individuals carrying E2+ were significantly lower (median: 89.0 [79.2–111.3]) than in individuals with E3/E3 genotype (median: 101.0 [82.0–121.0]), and also lower than individuals with E4+ (median: 105.0 [85.3–126.8]). In the analysis by genotypes (dividing individuals in five groups) only the HDL‐C levels showed statistically significant differences (Table 3), with HDL‐C levels: E3/E4 < E4/E4 < E3/E3 < E2/E4 < E2/E3.

**Table 2:** Summary of the biochemical characteristics of the MA population stratified by *APOE* alleles

	E2+ (*n* = 21)	E3/E3 (*n* = 1,589)	E4+ (*n* = 387)	*p*‐value
BMI^a^	26.8 (25.5–31.1)	27.1 (24.1–30.5)	27.1 (24.3–30.3)	.74
Normal weight	4 (19.05)	527 (33.17)	121 (31.27)	.61
Overweight	10 (47.62)	626 (39.40)	162 (41.86)	
Obese	7 (33.33)	436 (27.44)	104 (26.87)	
TG	160.0 (120.0–240.0)	166.0 (120.0–234.5)	179.5 (125.8–258.8)	.05
TC	171.0 (160.0–212.0)	177.0 (151.8–204.0)	181.0 (159.0–208.8)	.06
HDL‐C	**41.0 (38.3–51.8)**	**38.0 (31.6–46.0)**	**36.0 (30.0–44.0)**	**.001**
LDL‐C	**89.0 (79.2–111.3)**	**101.0 (82.0–121.0)**	**105.0 (85.3–126.8)**	**.02**
FG	92.0 (85.0–169.0)	92.0 (82.0–105.0)	91.5 (82.0–104.8)	.71

Note

In bold, biochemical variables that show statistically significant differences.

Abbreviations: BMI, body mass index; FG, fasting glucose; HDL‐C, high‐density lipoprotein cholesterol; LDL‐C, low‐density lipoprotein cholesterol; TC, total cholesterol; TG, triglycerides.

a For the biochemical variables, the results are reported as medians (interquartile range in parentheses).

**Table 3:** Summary of the biochemical characteristics of the MA population stratified by *APOE* genotypes

	*APOE* genotype					*p*‐value
	E2/E4	E2/E3	E3/E3	E3/E4	E4/E4	
BMI^a^	25.8 (25.2–27.3)	27.2 (26.4–30.7)	27.1 (24.1–30.4)	27.0 (24.2–30.2)	27.4 (24.7–30.8)	.80
TG	132.5 (97.75–480.0)	160.0 (132.0–231.0)	166.0 (120.0–234.5)	183.5 (125.0–261.0)	173.5 (131.8–257.2)	.21
TC	204.5 (165.0–271.8)	171.0 (158.5–196.5)	177.0 (151.8–204.0)	179.0 (158.0–210.0)	185.0 (160.0–206.0)	.18
HDL‐C	**39.5 (30.9–47.0)**	**41.0 (39.3–51.8)**	**38.0 (31.6–46.0)**	**36.0 (30.0–44.0)**	**37.0 (30.0–45.0)**	**.006**
LDL‐C	96.5 (81.1–127.3)	89.0 (77.5–111.1)	101.0 (82.0–121.0)	105.0 (85.5–126.5)	107.0 (85.0–127.5)	.09
FG	120.0 (95.0–205.0)	89.0 (76.5–134.0)	92.0 (82.0–105.0)	92.0 (82.0–105.0)	89.0 (78.0–102.0)	.23

Note

In bold, biochemical variables that show statistically significant differences.

Abbreviations: BMI, body mass index; FG, fasting glucose; HDL‐C, high‐density lipoprotein cholesterol; LDL‐C, low‐density lipoprotein cholesterol; TC, total cholesterol; TG, triglycerides.

a For the BMI and biochemical variables, the results are reported as medians (interquartile range in parentheses).

Second, we analyzed the biochemical characteristics after stratifying the population based on the BMI (normal with BMI <25, overweight with BMI ≥25 or <30 and obese with BMI ≥30). As the number of individuals carrying E2+ allele was very low, we only stratified and analysed individuals homozygous for E3 allele and individuals carrying E4+ (*n* = 1,976 individuals). In normal weight and overweight individuals, *APOE* alleles or genotypes did not show differences in any of the biochemical variables (Table 4). Also, the BMI in the individuals did not differ between the different *APOE* allele or genotype carriers (Tables 2 and 3). Meanwhile, obese individuals carrying E4+ allele had higher TC, TG, and LDL‐C values than individuals homozygous for E3 allele. In contrast, obese individuals carrying E4 allele (median: 34.0 [29.0–40.5]) had reduced HDL‐C levels when compared with obese individuals homozygous for E3 allele (median: 37.0 [31.0–44.8]). In the analysis by genotype (i.e. comparing E3/E3, E3/E4, and E4/E4), only HDL‐C levels remain with statistically significant.

**Table 4:** Summary of the biochemical characteristics of the MA population stratified by *APOE* genotypes

	All	E3/E3	E3/E4	E4/E4	E4+	*p*‐value^a^	*p*‐value^b^
Normal weight (BMI < 25.0 kg/m^2^)							
*n*	648 (32.79)	527 (81.33)	110 (16.98)	11 (1.70)	121 (18.67)	—	—
TG	146.0 (103.0–200.0)	143 (102.5–196.5)	156.0 (120.0–221.0)	133.0 (120.0–221.0)	155.0 (106.0–212.2)	.21	.46
TC	172.0 (147.0–199.0)	172.5 (147.0–198.0)	172.0 (145.0–202.0)	164.0 (158.0–199.0)	171.5 (146.0–201.8)	.85	.96
HDL‐C	40.0 (33.0–50.38)	40.0 (33.0–51.0)	39.0 (31.8–50.0)	38.3 (36.0–45.0)	39.0 (32.0–48.0)	.10	.24
LDL‐C	98.0 (80.0–116.5)	98.0 (80.0–116.0)	103.0 (82.0–117.0)	98.0 (89.0–137.0)	102.0 (82.5–118.2)	.40	.66
FG	90.0 (81.0–101.0)	90.0 (82.0–101.0)	90.0 (81.0–105.0)	89.0 (78.0–91.0)	90.0 (80.3–105.0)	.82	.69
Overweight (BMI ≥ 25.0 kg/m^2^ and <30.0 kg/m^2^)							
*n*	788 (39.88)	626 (79.44)	147 (18.65)	15 (1.90)	162 (20.56)	.40	.66
TG	182.0 (131.5–260.0)	182.0 (133.0–258.0)	188.0 (129.5–273.0)	200.5 (123.5–264.8)	188.0 (128.5–271.5)	.60	.85
TC	180.5 (157.0–207.0)	180.0 (155.0–206.0)	180.0 (162.0–210.0)	200.0 (173.5–225.0)	181.0 (162.0–210.0)	.27	.31
HDL‐C	36.9 (30.0–44.0)	37.0 (30.2–44.0)	35.9 (30.0–42.0)	36.8 (28.5–46.5)	36.0 (29.8–43.3)	.25	.4
LDL‐C	103.0 (85.0–124.0)	103.0 (85.0–122.3)	104.0 (85.0–132.0)	111.0 (99.8–134.0)	104.0 (85.0–132.0)	.23	.43
FG	92.0 (83.0–105.0)	92.0 (83.0–106.0)	92.0 (83.0–104.0)	88.0 (76.5–101.0)	92.0 (82.5–104.0)	.53	.50
Obese (BMI ≥ 30.0 kg/m^2^)							
*n*	540 (27.33)	436 (80.74)	95 (17.59)	9 (1.66)	104 (19.26)	.85	.96
TG	186.0 (134.0–247.0)	181.0 (132.0–241.0)	203.0 (141.0–292.0)	173.0 (150.0–223.0)	199.5 (146.0–289.5)	.02	.08
TC	181.5 (156.0–207.0)	180.0 (154.0–207.0)	186.0 (164.8–213.0)	184.0 (167.0–191.0)	186.0 (165.0–213.0)	.03	.06
**HDL‐C**	**36.0 (30.0–44.0)**	**37.0 (31.0–44.8)**	**33.5 (28.3–39.5)**	**34.0 (33.0–42.0)**	**34.0 (29.0–40.5)**	**.003**	**.01**
**LDL‐C**	**104.0 (83.3–123.1)**	**101.0 (82.0–122.0)**	**113.0 (96.0–126.0)**	**99.5 (77.8–120.8)**	**112.0 (92.0–126.0)**	**.03**	**.05**
FG	93.5 (83.0–106.8)	93.0 (83.0–107.0)	94.0 (82.8–104.3)	90.0 (78.0–102.0)	94.0 (82.0–104.0)	.93	.97

Note

In bold, variables that show statistically significant differences.

Abbreviations: BMI, body mass index; FG, fasting glucose; HDL‐C, high‐density lipoprotein cholesterol; LDL‐C, low‐density lipoprotein cholesterol; TC, total cholesterol; TG, triglycerides.

a *p*‐value reported for analysis by allele.

b *p*‐value reported for analysis by genotype.

Next, we performed an analysis of LDL‐C and HDL‐C, in the individuals with different grades of obesity (obesity grade 1 with BMI ≥30 or <35, obesity grade 2 with BMI ≥35 or <40 and obesity grade 3 with BMI ≥40). In this analysis, differences in LDL‐C remain significant only in the individuals with obesity grade 1, between individuals homozygous for E3 allele (median: 101.0 [83.5–119.5]) and individuals carriers of the E4 allele (median: 110.5 [92.75–126.0]) (*p*‐value = .04). Also, differences in HDL‐C remain significant only in individuals with obesity grade 2, both by alleles and by genotype of ' carriers of the E4 allele (median: 110.5 [92.75–126.0]) (*p*‐value = .04). Also, differences in HDL‐C remain significant only in individuals with obesity grade 2, both by alleles and by genotype of *APOE*.

## 4 DISCUSSION

The association of *APOE* alleles with lipid and glucose metabolism has been replicated in different populations worldwide (Baila‐Rueda et al., 2016; Breitling et al., 2015; Ward et al., 2009; Yasuno et al., 2012). Several studies have proposed that individuals have metabolic differences in lipid regulation depending on the expressed *APOE* alleles, and that some of pathological processes promoted by ApoE may correlate with isoform‐dependent changes at the structural level of the protein (Mahley et al., 2009). Also, these differences in the modulation of lipid profile depending on the ApoE isoform could be influenced by the variation in *APOE* alleles based on the ethnic background of the population analyzed. Therefore, in this study, we performed an analysis of the relationship between the *APOE* alleles and the biochemical profile in the MA population.

We compared the *APOE* allele frequency in our population with frequencies reported by Eichner et al. in a metanalysis of allelic and genotypic *APOE* frequencies, and we found that our MA population has some of the lowest E2 and E4 allele frequencies of the populations included in the meta‐analysis (Eichner et al., 2002). These differences of *APOE* allele frequency in the MA population could indicate different disease risks; for instance, the E4 allele that confers a higher risk of coronary artery disease (CAD) or AD, in the MA populations is less frequent and could be important in the modulation of lower risk for CAD or AD that has been previously reported for Hispanic populations (Fujiyoshi et al., 2017; Maestre et al., 1996).

Regarding the modulation of lipid profile by *APOE* in MA individuals, we found that MA carriers of E4+ allele had lower HDL‐C and higher LDL‐C levels than homozygotes for E3 allele, and then E2+ carriers. In agreement with our observations, differential modulation of lipid profile (HDL‐C, LDL‐C, and TC) dependent on the ApoE isoform has been reported previously in other populations (Bennet et al., 2007; Boulenouar et al., 2013; Volcik et al., 2006; Wu et al., 2007). The association between *APOE* alleles and lipid profile has been one of the most studied worldwide, and even these results have been validated in an animal model (Hopkins, Huang, McGuire, & Pitas, 2002).

It has been reported that the effects of *APOE* alleles on differential lipid profile modulation depend on both genetic and environmental factors (Bernstein et al., 2002; Nicklas et al., 2002). One factor that may modulate the interaction of lipid levels with ApoE is the BMI (Cronk, Johnson, & Burns, 2010; Gamboa et al., 2008; Romas, Tang, Berglund, & Mayeux, 1999). In the present study, obese individuals carrying the E4 allele had lower HDL‐C levels and higher TC and TG levels than obese individuals homozygous for E3 allele. A decrease of HDL‐C levels has been consistently associated with an increased risk of CAD, increased mortality, atherosclerosis and decreased cognitive function (Christiansen et al., 2017; Gillum & Obisesan, 2011; Larifla et al., 2017). The CAD risk is inversely dependent on HDL‐C plasma levels (DeFaria Yeh, Freeman, Meigs, & Grant, 2007; Mahdy Ali, Wonnerth, Huber, & Wojta, 2012). Based on the relationship found in our analysis in which the E4 allele promoted low HDL‐C levels, this effect was potentiated in obese MA individuals. In the MA population it has been reported a higher prevalence of obesity (Hu, Huff, Yamamura, Wu, & Strom, 2015; Schulz & Chaudhari, 2015) compared to other populations; thus, the high incidence of obesity in the MA population places the MA population at a high risk for the development of CAD.

The MA population has been neglected throughout history in terms of being exposed to factors that affect their health. Therefore, the present work is one of the first analyses of possible factors that can modulate lipid profiles in the MA population. In another study, were only 146 MA individuals were included, it was found an association between the E2/E3 genotype and low levels of LDL‐C (Aguilar et al., 1999). On the other hand, although one of the strengths of the present study is the moderate sample size of MA individuals included, we consider as a limitation the lack of endophenotype data that may help to understand the association between *APOE* polymorphism and other biochemical markers in this population (Bojar et al., 2015; Smalinskiene et al., 2013). Nevertheless, the results are interesting and further investigations could lead to new knowledge in the way genetic variants affect the modulation of lipid profile in MA populations.

## 5 CONCLUSIONS

The analysis of this cohort of MA ancestry individuals support findings observed in other populations, showing that individuals with *APOE‐E4* polymorphism present lower HDL‐C and higher LDL‐C, TG and TC levels. Additionally, the effect of the E4 allele decreasing HDL‐C levels, is enhanced in obese MA individuals.

## ETHICS APPROVAL AND CONSENT TO PARTICIPATE

All procedures performed in this study were carried out in accordance with the Helsinki declaration. The protocol was submitted and approved by the ethical and research committees of the National Institute of Genomic Medicine (number INMG/DI/149/2014). Written informed consent was obtained from all subjects included in the protocol.

## CONFLICT OF INTEREST

The authors declare that they have no competing interests.

## AUTHORS' CONTRIBUTIONS

JJMM, ADGM, CATZ, TBGC, and YHD contributed to the writing of this manuscript/ equally to this research. AMH, HGO, and LO conducted the laboratory and data analyses. IJR, MLLN, and HN interpreted the data and drafted the initial manuscript. All authors revised the initial manuscript. All authors approved the final draft.
